# Pharmacokinetics study of chitosan-coated liposomes containing sumatriptan in the treatment of migraine

**DOI:** 10.22088/cjim.13.1.90

**Published:** 2022

**Authors:** Sara Assadpour, Javad Akhtari, Mohammad Reza Shiran

**Affiliations:** 1 Molecular and Cell Biology Research Center, Faculty of Medicine, Mazandaran University of Medical Sciences, Sari, Iran; 2 Department of Medical Nanotechnology, School of Advanced Technologies in Medicine, Mazandaran University of Medical Sciences, Sari, Iran; 3 Toxoplasmosis Research Center, Communicable Diseases Institute, Faculty of Medicine, Mazandaran University of Medical Sciences, Sari, Iran; 4 Department of Pharmacology, Faculty of Medicine, Mazandaran University of Medical Sciences, Sari, Iran

**Keywords:** Chitosan, Nanoliposome, Sumatriptan succinate, Migraine, Bioavailability

## Abstract

**Background:**

Sumatriptan is a routine medication in the treatment of migraine and cluster headache that is generally given by oral or parental routes. However, a substantial proportion of patients suffer severe side effects. The aim of this study was to investigate the physicochemical characterization and pharmacokinetic parameters of a novel delivery system for sumatriptan succinate (SS) using nanoliposomes (NLs) coated by chitosan (CCLs) to optimize the formulations to enhance its bioavailability.

**Methods:**

The new formulation was used to minimize drug particle size and extend its release and bioavailability. The mean particle size and entrapment efficiency for NLs and CCls were optimized and the formulations with better characteristics were chosen for *in vivo* studies. The concentration-time profile of intravenous SS, intranasal SS, SS-NLs, and CCLs were examined in a rabbit model.

**Results:**

The results demonstrated that CCLs were absorbed more rapidly from nasal drops containing chitosan compared to those of SS and SS-NLs as indicated by a shorter t_max_, and a higher C_max_ in both states. A comparison of the AUC (0-240 min) values revealed that chitosan improved the extent of SS absorption for CCLs formulation. The results of the present study indicated that loading SS into the liposome and coating with chitosan improves drug absorption and a large amount of the drug can be efficiently delivered into the systemic circulation.

**Conclusion:**

The liposomal and chitosan formulations of SS had better kinetic behavior than the soluble form in the animal model.

Migraine is a common and highly debilitating condition that has substantial social and economic burdens (1). This condition affects approximately 10-15% of the general population in Iran and worldwide (2, 3). Migraine also is more frequent among women and those aged 30-40 years (4). Migraine is initially a unilateral head pain disorder with recurrent, throbbing, and intense features. Associated manifestations include nausea, vomiting, photophobia, and sensitivity to smell or sound (5). The exact underlying pathogenic mechanisms of migraine attaches still remain unclear. In the past decade, a growing body of evidence accumulated from preclinical and clinical data has confirmed that both vascular and neuronal structures are involved via local intracranial and extracerebral vasodilation of blood vessels and simultaneous induction of pain cascades surrounding the trigeminal sensory nerve leads to migraine attacks (6). The generation of pain in migraine attack is related to the neurogenic inflammation, production of neuropeptide, and activation of meningeal afferents (5). 

A wide range of therapeutic approaches for the treatment of migraine exists for many years with varying modes of action and differing degrees of efficiency (7). The drugs, such as analgesics, NSAIDs, corticosteroids and triptans are prescribed in acute migraine (8). Due to the prominent role of serotonin (5-hydroxytryptamine [5-HT]) in the improvement of migraines and the presence of serotonin receptors on the trigeminal nerve and cranial vessels, the triptans, a class of synthetic drugs selective serotonin receptor agonists was developed (9). Sumatriptan (ST) with various routes of administration (oral, intravenous, rectal, and intranasal) was the first triptan to be licensed for the treatment of adults with migraine (10). 

The analgesic effects of ST and its salt, sumatriptan succinate (SS) probably are related to the induction of serotonin receptors: 5-HT1B receptor activation results in vasoconstriction, and 5-HT1D receptor stimulation inhibits the dural neurogenic inflammation, and its activation in brainstem suppresses the sensitization in the trigeminal nuclei (11). 

The intranasal delivery system of drug has been proposed for the treatment of various neurological conditions, e.g., sleeping disorders, brain tumors, schizophrenia, multiple sclerosis, Parkinson’s disease, Alzheimer’s disease, and migraine (12). Non-invasive intranasal administration of drugs has been considered in clinical studies due to its effectiveness as a result of the easy access to the nasal mucosa with the rich vascular supply (13), and the rapid achievement of effective pharmacokinetic levels, possibly through first-pass hepatic metabolism circumvention (14). As migraine has a high association with nausea and gastrointestinal symptoms that might lead to unreliable absorption of oral medications, the intranasal route of administration has theoretical advantages and is less invasive than subcutaneous or intravenous routes of medication delivery (15). However, some anatomical and physiological limitations, including the small volume nasal cavity, mucosal irritation, lower deposition of therapeutic agents deposit from conventional delivery devices (pumps and sprays) in the olfactory region, reduced residence time, and lower transportation of large hydrophilic molecules, may negatively affect nose-to-brain transportation of drugs (16, 17). Therefore improving the drug delivery systems of SS can help to enhance anti-migraine potential. 

Recently, several drug delivery systems, have aimed to enhance effectiveness and decrease the toxic properties of the active compounds, have been developed for the treatment of neurological diseases (18). The blood-brain barrier (BBB), a unique vital element of microvasculature  in the central nervous system (CNS), which can selectively transport the molecules from the blood to the brain should be considered in the establishment of drug delivery systems for treatment of neurological disorders (19). Last decades, the development of nanoparticles (NPs) with medical purposes, especially as carrier systems of drugs, attracts more attention(20). Liposomes, nanoparticle platform applied in medicine, and particularly nanoliposomes (NLs) are one of the most used NP-based systems for delivery of small molecules, like peptides, nucleic acids, and proteins (21). Chitosan, an FDA approved substance abundantly found in nature, also is a non-toxic, biodegradable, and non-immunogenic polymer (22). The positive groups of NPs prepared with chitosan are suitable forms of carries in intranasal delivery systems for therapy of brain diseases due to their mucoadhesive nature which can attach to negatively charged mucosal secretions of the nasal cavity (23).

The aim of present study was to investigate the physicochemical characterization, and pharmacokinetics parameters of a novel delivery system for sumatriptan succinate (SS) using nanoliposomes (NLs) coated by chitosan (CCLs) to optimize the formulations to enhance its bioavailability through the nasal route.

## Method

White New Zealand male rabbits (2.5±0.2Kg) were prepared from Pasteur Institute of Iran. SS (99.2%) was obtained from supplied from Natco Fine Pharmacis Pvt Ltd. Hyderabad, Hydrogenated soya phosphatidylcholine (HSPC) were purchased from Lipoid (Ludwigshafen, Germany. Amicon centrifugal filter device (Amicon®Ultra-4, Millipore) were used prepared. Drug-free human plasma was prepared from Biological Specialty Corporation (Colmar, PA, U.S.A.). Acetonitrile, methanol, ethyl acetate, sodium phosphate, formic acid, and sodium hydroxide were purchased from Merck KGaA (Germany). Chitosan with a molecular weight of 150 kDa and a degree of deacetylation (76%) and a purity of 99.98% was purchased from Sigma-Aldrich) USA). Transmission electron microscopy (TEM) (JME2010 TEM, JEOL) was used. In our investigation, all other reagents were of analytical grade and are utilized without further purification. Ethical approval (IR.MAZUMS.REC.1396.2827) was obtained from the Ethics Committee of Mazandaran University of Medical Sciences (Grant number: 2827).

**Preparation of nanoliposomes: **To prepare SS for NL formulation, SS was solubilized in dextrose (5%). Liposomes were prepared via thin film hydration and extrusion method (24). In summary, chloroform stock solutions were prepared via adding appropriate amounts of cholesterol and phospholipids in a round bottom balloons. Under vacuum, it was subjected to solvent evaporation in a rotary evaporator followed by overnight freeze-drying. Solution of SS (250 mM) was added to thin film to obtain final phospholipid concentration of 11 mM, then, sonicated for 10 min under argon atmosphere (60°C). Next, polycarbonate nanopore membranes of 80, 100 and 200 nm pore size (Avestin, Canada) were used to extrude the newly formed NLs. To purify the prepared NL formulations containing SS from the unencapsulated one, the liposomes were dialyzed three times dextrose (5 %) sucrose in dialysis cassettes (Pierce, Rockford, IL) with 12 to 14 kDa molecular weight cutoff (MWCO). 

**Coating of nanoliposomes with chitosan: **To coat NLs and make new formulation, chitosan-coated liposomes (CCLs), NLs suspension (in 5 % dextrose) were added drop wise into the 0.001% chitosan solution (w/v) (in 0.1 M acetic acid). Dilute NaOH was added to adjust the pH of suspension (4.5) under stirring (250 rpm at 20–22 °C) in a volume ratio of 1:4. The suspension was stored overnight at 20–22 °C. CCLs were isolated from the mixture via centrifugation technique (15,000×g) for 30 min at 4 °C and resuspended in dextrose 5 % saline. This washing procedure was done twice.

**Evaluation of SS-NLs and CCLs characteristics**

***Transmission electron microscopy (TEM) and Dynamic light scattering: *** The prepared formulations’ shape and formation were also evaluated by TEM. For this purpose, the samples were fixed on a carbon-coated copper grid and the images were prepared by a LEO 912AB Omeg TEM (Zeiss, Jena, Germany) at a 120 kV acceleration voltage. Zeta potentials of NLs and CCLs were evaluated using an electrophoretic light scattering using Malvern Zetasizer Nano ZS. 

***In vivo studies: *** The experimental protocol for all in vivo studies was approved by the Animal Ethics Committee of Mazandaran University of Medical Sciences, Sari, Iran. New Zealand white male rabbits (mean weight of 2.5±0.3 kg) provide a well-controlled animal model for screening the nasal absorption potential of nasal delivery of formulations (25). About 2 weeks before the study, the rabbits were acclimatized. The animals were fasted for 15-17 hours prior to drug administration with free access to water. The rabbits were allocated in four groups as followed: 

**SUMA1:** SS solution (20 mg/ml) was intravascularly injected.

**SUMA2:** SS solation (20 mg/ml) was intranasally administered via a polyethylene tube.

**NL: **NLs containing SS (20 mg/ml) was intranasally administered via a polyethylene tube.

**CCL: **CCLs containing SS (20 mg/ml) was intranasally administered via a polyethylene tube.

All nasal preparations were administered via a polyethylene tube into each nostril while the rabbit’s head was held in an upright position so that a total volume of 250 µL per rabbit was administered. The tube tip was inserted 5 mm into the nostril and the rabbit was kept in this position for 1 min after the administration to prevent leakage of the preparation out of the nostril. Usual anesthetizing agents could affect the *in vivo* results of SS absorption from different delivery systems. Therefore, to avoid any influence of the anesthesia in the SS absorption as well as providing functional mucociliary transport throughout the procedure, rabbits were kept conscious during the experiments and were permitted to breathe normally through the nostrils. After the administration of drugs in different groups, absolute bioavailability of SS was calculated in different time points. Blood samples were withdrawn from the marginal ear vein at 15, 30, 45, 60, 90, 120, 180, 240 min after administration. Plasma concentrations of ST were determined via HPLC system (26). 

***Extraction procedure for plasma samples: *** Firstly, 0.5 ml aliquot of rabbit plasma sample was placed in a screw cap glass tube. Then, 0.5 ml of 1 M sodium hydroxide solution was added and the mixture was vortexed for 3 s. The rabbit plasma samples containing SS were then extracted with 3.4ml of ethyl acetate. The mixture was shaken for 10 min and centrifuged for 10 min at room temperature. The extract was transferred to a culture tube, and then evaporated to dryness under a nitrogen stream. The extraction residue was reconstituted in 0.20 ml of the mobile phase, then, injected into the HPLC system (27, 28).

R**ecovery**: The absolute recovery of SS was assessed by calculating the peak area of extracted sample. The overall recovery of SS was 88.5% (27).

***Pharmacokinetic analysis: *** The area under the concentration time curves (AUC) from dosing to the time point (tn) of the last non zero value (AUC 0-tn) was calculated by the linear trapezoidal method. The C_max_ and t_max_ values were reported visually.

***Data Analysis: *** All formulations were prepared and analyzed in triplicate. Results are expressed as mean ± SD. A linear analysis of variance model (ANOVA) was used to analyze the AUC, C_max_ T_max_ and the results were obtained from pharmacokinetic studies. Statistical significance was considered as p ≤ 0.05. Data analyses were performed with GraphPad Prism® software, version 5.01.

## Results

**Fig 1:**
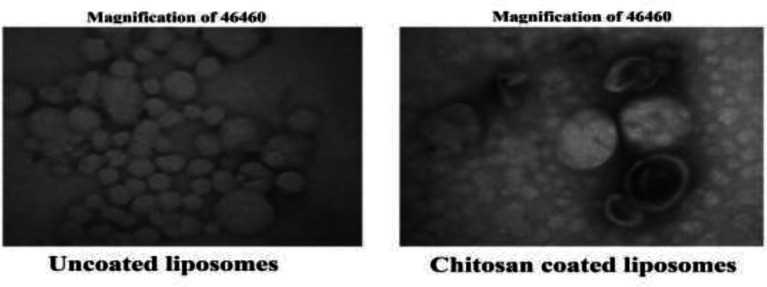
TEM images of liposomes containing SS with spherical shape. Uncoated liposomes at magnifications ×46460. Chitosan-coated liposomes at magnifications ×46460.

***Characteristics of liposomes by TEM: *** By TEM evaluations, the spherical form and the narrow size distribution of liposomes were confirmed. Both chitosan-coated liposomes and uncoated liposomes had spherical shape (figure 1) and there were no significant differences between the sizes of chitosan-coated liposomes and uncoated liposomes results, there was a significant difference between the size of NLs (142.33±8.38nm) and CCLs (167.33±3.39nm) liposomes (P=0.0001). 

**Fig 2:**
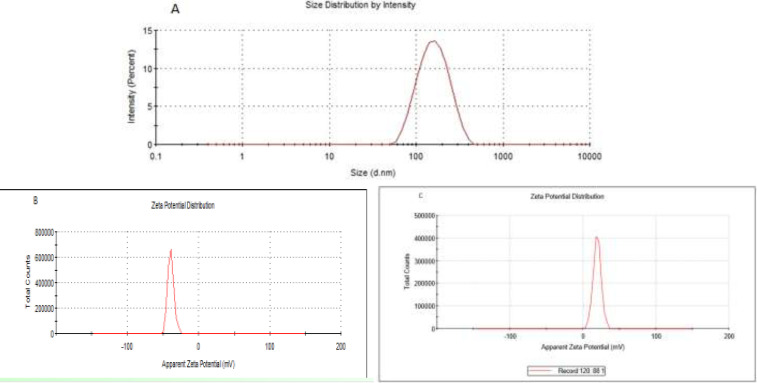
Particle size and zeta potential distribution of nanoliposomes. A: size distribution of nanoliposomes: 155 nm. B: Zeta potential liposomes before attachments of chitosan. C: Zeta potentials of chitosan-coated liposomes

***Entrapment efficiency and physicochemical studies of drug release:*** The average drug entrapment efficiency rates of SS-NL and CCLs were 22.3±2.7 and 21.5±2.1%, respectively. According to the drug-polymer ratio, the cumulative drug release for SS-NL was 59.4±1.1% for 24 hours and for CCL formulation was 75.2± 1.2% for 24 hours. The standard graph of SS absorbance based on its concentration and its formula was calculated in fig 1-A. According to the results, the calculated concentration increased with the increasing percentage of SS. Significant differences were observed between 10% and 2, 1 and 0.5% concentrations (P=0.0001). The calculated concentrations at 2% were reported to be significantly higher than SS 0.05 and 1% groups (P=0.0001). Also, the calculated concentration was significantly higher than 0.05% SS (P=0.0001). 

**Fig 2:**
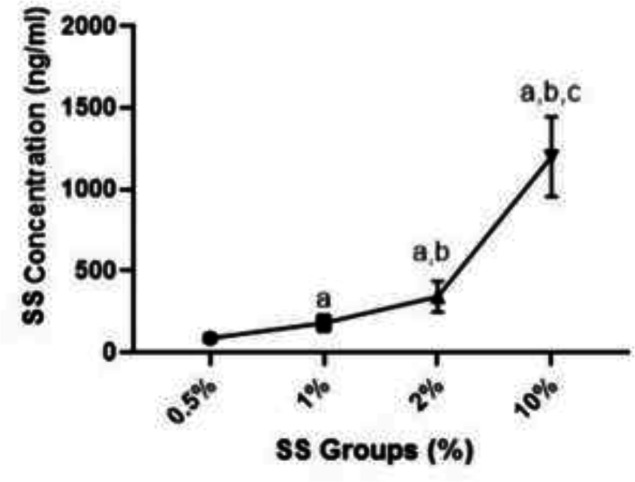
A) Chromatogram of SS based on different doses. a Significant difference with SS group 0.5% (p<0.05), b Significant difference with SS group 1% (p<0.05), c significant difference with SS group 2% (p<0.05)

***In vivo study: *** The pharmacokinetic parameter for AUC, C_max_ and t_max_ of different formulations of SS are detailed in table 1.

**Table 1:** Summary statistics for pharmacokinetic parameters of different formulations of sumatriptan (SS). SUMA1: SS solution after for IV injection, SUMA2: SS solution for nasal delivery, NL: nanoliposomes (NLs) containing SS for nasal delivery, CCL: Chitosan coated liposomes (CCLs) containing SS for nasal delivery

**Parameters**	**SUMA1**	**SUMA2**	**NL**	**CCL**
AUC_0- 240_ (ng.min/ml)	14860/8	1051/5	3478/8	5575/9
AUC_0-60_ (ng.min/ml)	10388/3	363/5	1220/8	2419/3
AUC_0- _90 (ng.min/ml)	12058/8	544/0	1733/3	3234/3
C_max_ (ng/ml)	234/0	6/4	17/7	30/8
t_max_^ b ^(min)	15	90	90	60

All nasal preparations were administered via a polyethylene tube into each nostril while the rabbit’s head was held in an upright position so that a total volume of 250 µL per rabbit was administered. The tube tip was inserted 5 mm into the nostril and the rabbit was kept in this position. The results demonstrated that CCLs were absorbed more rapidly from nasal drop containing chitosan compared to those of intranasal SUMA2 and NLs as indicated by a shorter t_max_, and a higher C_max_ in both states. A comparison of the AUC (0-240 min) values revealed that chitosan significantly (p<=0.01) improved the extent of SS absorption for CCLs formulation.

According to fig 1, plasma levels of SS in the form of intravenous administration was significantly higher than the nasal routes (p<= 0.01)). For the pharmacokinetic parameters AUC _0-240_ the 90% confidence limits for the mean ratios for CCLs compared to other formulation were not within the limits of 0.8 to 1.25 indicating that the formulations were not bioequivalent with respect to these parameters. Relative AUC_0-240_ C_max_ and t_max_ and their confidence intervals of different nasal formulations of sumatriptan (SS) are presented in table 2. 

**Table 2:** Relative AUC_0-240_ C_max_ and t_max_ and their confidence intervals of different nasal formulations of sumatriptan (SS). SUMA2: SS solution, NL: nanoliposomes (NLs) containing SS, CCL: Chitosan coated liposomes (CCLs) containing SS

**Comparison of** **Formulations**	**Mean ratio AUC**_0-240_	**Mean ratio C**_max_	**Median differencet**_max _ **(min)**
	**(90% CI)**	**(90% CI)**	**(90% CI)**
CCL/ NL	1.60 (1.13,2.12)	1.75 (1.27,2.24)	-30 (-27,-33)
CCL / SUMA2	5.30 (4.55,6.10)	4.84 (4.30,5.38)	-30 (-27,-33)
NL / SUMA2	3.31 (2.76,3.85)	2.77 (2.19,3.37)	0 (1,1)

Mean SS serum concentration *vs* time for each of study formulation are shown in figure 1 (all nasal and iv formulation upper panel) and (nasal formulation, lower panel).

**Figure 3:**
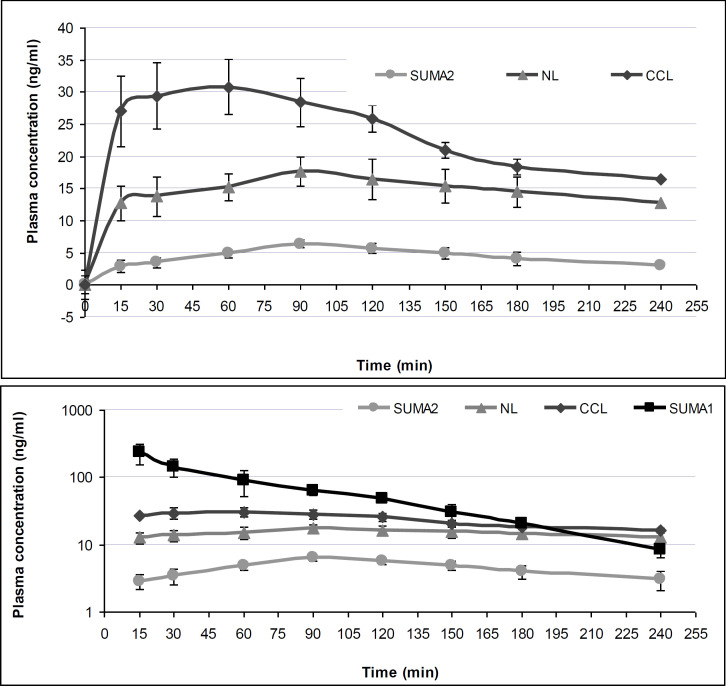
Mean (±SD) serum concentrations for three different nasal formulation of sumatriptan succinate (SS) in rabbit.SUMA2: SS solution, NL: nanoliposomes (NLs) containing SS, CCL: Chitosan coated liposomes (CCLs) containing SS

## Discussion

In the present study, the physiochemical characteristics and pharmacokinetics parameters of the novel delivery system for sumatriptan succinate (SS) using nanoliposomes (NLs) coated by chitosan (CCLs) to optimize the formulations to enhance its bioavailability, were evaluated. Although oral route of SS is widely used for treatment of migraine attacks, it can incompletely be absorbed and undergoes pre-systemic metabolism with lower bioavailability (15%)(29). Nausea and vomiting may follow oral administration of SS. Therefore the use of intravenous and intranasal routes have been recommended (30). In Iran the intranasal systems of SS are not available. On the other hand, the intranasal administration of SS via conventional devices leads to  poor olfactory region deposition and reduces the bioavailability and efficacy of drug (16). Therefore, we decided to develop novel drug delivery systems using nanoparticles to effectively enhance the therapeutic characteristics of SS by increasing the absorption of SS into the blood circulation. Such systems are effective strategies to improve the anti-migraine characteristics of SS (31). In the present study, we used NLs containing SS to deliver drug via nasal route to increase its concentration in blood. NLs have been developed to drug delivery because the methods of preparation are generally simple and easy to scale-up. NLs are self-assembling colloidal structures comprising of lipid bilayers encompassing an aqueous compartment, and can typify the wide range of hydrophilic drugs within this compartment (32). NLs have been demonstrated to provide stable epitome to different drugs and offer unique focal points over un-encapsulated agents (33). 

In the next step, to enhance the absorption of NLs from nasal mucus, we coated the NLs with chitosan. Chitosan, one of the most abundant biopolymers, is a natural cationic polymer with higher biodegradability, biocompatibility, bioactivity, and mucoadhesion characteristics (34, 35). Chitosan NPs in different shapes have been successfully used as drug delivery systems to improve the efficacy of several drugs (36-38). Due to its structure with native amine groups, chitosan is able to load hydrophobic and hydrophilic drugs in drug delivery systems (39). 

In the present study, we designed NLs and CCLs to enhance the bioavailability of SS. For the first step, the NLs containing SS were prepared and to enhance the mucoadhesion of negatively charged NLs, they were coated by positively charged chitosan. The aim of this strategy was to enhance adhesive characteristics of SS to the mucus of nasal cavity with negative electric charge to increase the chance of absorption via nasal vascular system, leading to the increased bioavailability of SS and maximum bioavailability. In the present study, the size of prepared SS-NLs and CCLs were 142.33±8.38 nm and 167.33±3.39 nm, respectively. In addition, both introduced nanoparticles in this study showed high drug release ability and entrapment efficiency. However, CCLs exerted superior features compared to NLs. The smaller size of nanoparticles enhances the penetration of biological membranes and increases drug concentration in targeted tissue (40). 

In line with the present study, SS loaded chitosan NPs was used as drug delivery systems to enhance the drug concentration in circulation. The results showed that using such systems could improve the entrapment efficiency (59.60±2.12%) and transport percentage (DTP) and drug targeting efficiency (DTE) were reported 79.79% and 493.39%, respectively (41). Therefore, using chitosan similarly improved the physicochemistry characteristics of SS containing NPs*.*

The *in vivo* studies on rabbits showed that the concentration of SS in the blood samples increased immediately after intravenous administration. On the other hand, the higher bioavailability of SS following nasal administration of both novel nasal delivery systems NLs and SS-CCLs was higher than conventional use of SS. Due to the shorter t_max_, and higher C_max_ in both state, CCLs were absorbed more rapidly from nasal drop due to its chitosan component compared to those of SS and SS loaded liposomes. Moreover, AUC (0-240 min) values indicated that chitosan improved the extent of SS absorption for CCLs formulation. Therefore, a large amount of the drug can be efficiently delivered into the systemic circulation via loading SS into the liposome and coating with chitosan and consequently, drug absorption and kinetic behavior were improved. 

Several studies have aimed to enhance the efficacy and bioavailability of the different routes of SS. In agreement with our study, in an experimental study, oral administration of chitosan solid lipid NPs containing SS showed significantly exerted anti-migraine activity via enhancing the blood concentration and crossing the BBB (20). The encapsulation of drug into the NPs such as chitosan increases the drug bioavailability mucoadhesion with higher characteristics, thus improving the drug dissolution in the mucosa (42). The higher efficiency of chitosan- based intranasal formulations are attributed to their higher ability to adhere on mucosal surfaces. These features can improve the dissolution of the drug in the mucosa with aqueous environment (43). In addition, it has been reported that NPs with bio-adhesive characteristics have a critical impact on the uptake of drugs from mucous membranes including the nasal (44). As absorption enhancers, chitosan and its derivatives are able to efficiently improve the nasal bioavailability and enhance the concentration of drugs in the circulation (45). Several studies have been trying to use chitosan-based NPs to enhance the efficiency of drugs. In a recent study, SS-loaded chitosan NPs have been used to improve the therapeutic effect of this drug. The formulation was optimized via Taguchi method design. A positive zeta potential and suitable entrapment efficiency were obtained (23). In another study, rivastigmine loaded chitosan NPs showed higher bioavailability and enhanced the absorption of drug in circulation via intranasal delivery. Chitosan had an essential role in drug loaded NPs uptake (46). 

 Additionally, a novel intranasal delivery system for SS, freeze-dried k-carrageenan/chitosan polyelectrolyte complex-based was reported to have the highest water uptake ability and mucoadhesive potential and provided a more controlled release of SS (47). 

It has been revealed that coating the surface of the liposomes with chitosan increases their stability (48). CCL was also observed to use the delivery of other drugs. Recently, the flexible LP coated by chitosan has been used for delivery of docetaxel. This novel approach could increase the anticancer feature and bioavailability of docetaxel (49). In another similar study, the dry-powder formulation of CCL for nasal delivery of ghrelin was demonstrated to have higher ability of adhesion to mucins, entrapment efficiency, productivity against trypsin, and lower storage degradation (50). Taken together, CCLs had effective characteristics to intranasal delivery of SS compared to NLs, suggested to be used to improve the characteristics of intranasal SS. 

## Conclusion:

In this study, a novel designed NL and CCL formulations were evaluated for their physicochemical and bioavailability studies. At first, prepared NLs containing SS could effectively improve the characteristics of SS and increase its bioavailability in animal models after intranasal administration. Secondly, we used chitosan to coat the NLs, change its charge, and subsequently enhance its stability and adhesion ability to the nasal mucus. Coating the drugs with this agent can help to increase the effectiveness of drug delivery systems. According to the findings of this research, NL and CCL formulations containing SS were stable and improved the pharmacological characteristics and bioavailability of SS delivery via nasal route. In the future, these formulations can be used in preclinical and clinical studies for the nasal administration of SS. As it has advantages, the patient himself is able to use the drug easily and also this method is non-invasive. Also, the circulation time of the formulations was longer than the free drug in the blood, which indicated that the drug remained in the nanoparticles and sustained release. Generally, further studies are needed to evaluate the efficacy of these intranasal systems in crossing the drugs from biological membranes such as nasal and their concentrations in the blood. 

## Funding:

This study was a part of Sara Assadpour’s Ph.D. dissertation and financially supported by Molecular and Cell biology Research Center (MCBRC), Faculty of Medicine, Mazandaran University of Medical Sciences, Sari, Iran, a grant number of 2827 and with ethical code IR.MAZUMS.REC.1396.2827

## Conflict of Interests:

There is no conflict of interest.
